# National health institutions and the management of COVID-19 pandemic: fulfilling the sustainable development goal three (SDG-3) in Nigeria

**DOI:** 10.1186/s12889-026-28896-8

**Published:** 2026-08-07

**Authors:** Gideon Ibukuntomiwa Folorunso, Moses M. Duruji, Felix C. Chidozie

**Affiliations:** Department of Political Science and International Relations, Covenant University, Ota, Ogun State Nigeria

**Keywords:** COVID-19 pandemic, Nigeria Centre for Disease Control (NCDC), Public health management, National health institutions, Multi-sectoral collaboration, Pandemic preparedness, Sustainable Development Goal 3 (SDG-3)

## Abstract

**Background:**

The Coronavirus Disease 2019 (COVID-19) pandemic posed significant challenges globally, testing the capacity of national health institutions to respond effectively. In Nigeria, the pandemic revealed systemic gaps but also demonstrated institutional resilience. This study examines the broader institutional responses to the pandemic in Nigeria, involving the Nigeria Centre for Disease Control and Prevention (NCDC), the Presidential Task Force (PTF), the West African Health Organisation (WAHO), and state-level bodies, and their contribution toward achieving Sustainable Development Goal (SDG) Target 3.d, which focuses on strengthening national capacity for early warning, risk reduction, and management of national and global health risks.

**Methods:**

A retrospective qualitative research design was employed. In-depth interviews were conducted virtually via teleconferencing with seven purposively selected top-level directors from the NCDC, Nigerian Institute of Medical Research (NIMR), WAHO, and the Lagos State COVID-19 Case Management and Primary Health Care teams. Data saturation guided the sample size. Thematic analysis was guided by the WHO Framework for Strengthening Health Systems (2007) and reported in accordance with the COREQ checklist.

**Results:**

Findings reveal that the national response was initially hampered by severe challenges, primarily limited testing capacity, inadequate healthcare infrastructure, and widespread misinformation. In response, a multi-sectoral collaboration between federal and state governments, the private sector (e.g., CACOVID), and international partners was forged to expand diagnostics and enforce public health measures.

**Conclusions:**

Nigeria’s institutional response to COVID-19 highlights the importance of robust, coordinated national health frameworks. Strengthening Nigeria’s health system through sustained investment, public-private partnerships, and proactive risk communication is essential to fulfilling SDG Target 3.d and enhancing future pandemic preparedness.

**Supplementary Information:**

The online version contains supplementary material available at https://doi.org/10.1186/s12889-026-28896-8.

## Introduction

**Fig. 1:**
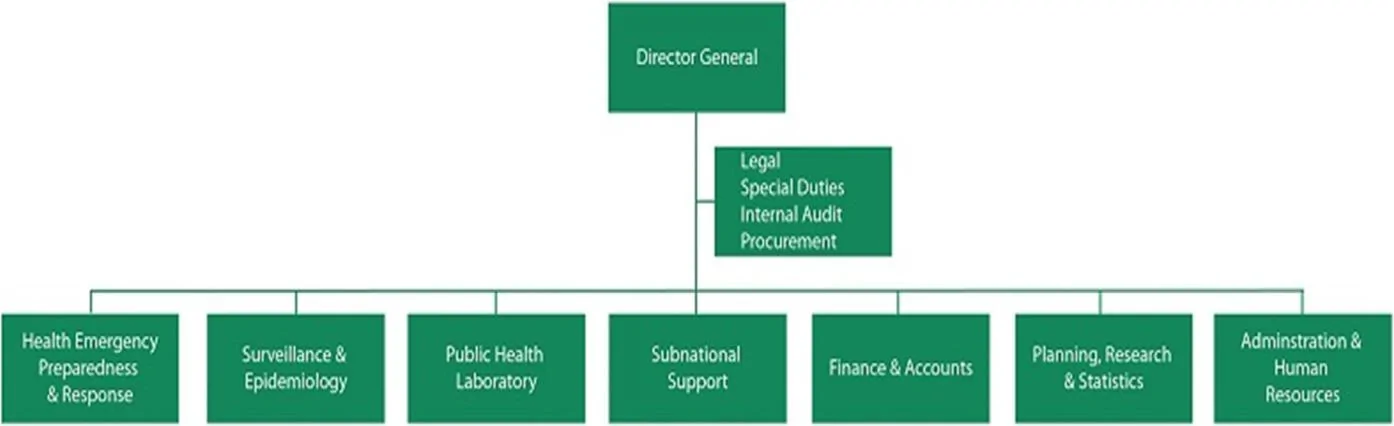
NCDC Organogram [4]

The institutions of government charged with the responsibility to ensure that the health of the population is cared for and secured are known as National Health Institutions (NHIs). While NHIs perform diverse functions ranging from health insurance management to hospital board administration, specific bodies are saddled with the core function of infectious disease management. In Nigeria, the Nigeria Centre for Disease Control and Prevention (NCDC) (see Fig. 1 for the organizational structure) and the Nigerian Institute of Medical Research (NIMR) play this pivotal role. Globally, the management of infectious disease outbreaks is the responsibility of the World Health Organisation (WHO), which further delegates duties to member countries through International Health Regulations. 

This study aims to answer the following research questions: (1) What specific actions and collaborations did national and international health institutions undertake in response to the COVID-19 outbreak in Nigeria? (2) What main challenges militated against these management efforts? (3) What measures have been put in place to ensure national pandemic preparedness for future outbreaks? By answering these questions, the study evaluates how these institutional responses align with Sustainable Development Goal (SDG) Target 3.d, which mandates the strengthening of national capacity for early warning, risk reduction, and the management of national and global health risks.

During the COVID-19 pandemic, the steering of the national response was handled by the Presidential Task Force (PTF) on COVID-19. Unlike the NCDC, which is a permanent statutory public health institute established in 2011, the PTF was an ad-hoc, high-level multi-sectoral coordinating body established by President Muhammadu Buhari on March 9, 2020 [1]. The PTF comprised representatives from various ministries, including health, environment, housing, security, agriculture, and information, to oversee Nigeria’s inter-governmental efforts. Building on the lessons learned during the successful containment of the 2014 Ebola outbreak, public health officials were able to leverage existing strategies and technologies for the COVID-19 response [2]. Early non-pharmaceutical interventions, including travel restrictions enacted on March 18, 2020, supervised quarantine, and dusk-to-dawn curfews, were rapidly deployed by the government to break the transmission chain [3].

To improve national testing capacity early in the pandemic, the NCDC and partner agencies rapidly expanded the number of public laboratories capable of testing for COVID-19 from five to twenty-three. Despite these efforts, disease management faced resistance. For instance, several faith-based organisations and individuals defied public health directives on social distancing by continuing to conduct large congregational services. To cushion the economic shock and fund the health response, Nigeria secured rapid COVID-19 recovery loans of US3.4 billion from the International Monetary Fund (IMF) during the first half of 2020. To illustrate the extent of the outbreak that necessitated these drastic public health and economic measures, a summary of confirmed COVID-19 cases and fatalities in Nigeria is presented in Table 1.

**Table 1:** Confirmed COVID-19 cases by state in Nigeria as of October 2022

State	Confirmed cases (lab confirmed)	Cases (Admitted)	Discharged	Deaths
Lagos	104,286	1143	102,372	771
FCT	29,535	9	29,277	249
Rivers	18,112	-3	17,960	155
Kaduna	11,675	3	11,583	89
Plateau	10,365	4	10,286	75
Oyo	10,352	0	10,150	202
Edo	7928	0	7606	322
Delta	5858	576	5170	112
Ogun	5810	11	5717	82
Kano	5429	11	5291	127
Ondo	5173	315	4749	109
Akwa Ibom	5010	6	4960	44
Kwara	4691	452	4175	64
Gombe	3313	8	3239	66
Osun	3311	29	3190	92
Enugu	2952	13	2910	29
Nasarawa	2853	469	2345	39
Anambra	2825	46	2760	19
Imo	2691	3	2630	58
Ekiti	2466	0	2438	28
Katsina	2418	0	2381	37
Benue	2317	88	2204	25
Abia	2263	0	2229	34
Ebonyi	2064	28	2004	32
Bauchi	2028	2	2002	24
Borno	1629	5	1580	44
Taraba	1517	32	1451	34
Bayelsa	1373	2	1343	28
Adamawa	1312	134	1140	38
Niger	1183	165	998	20
Cross River	947	0	922	25
Sokoto	822	0	794	28
Jigawa	669	2	649	18
Yobe	638	4	625	9
Kebbi	480	10	454	16
Zamfara	375	0	366	9
Kogi	5	0	3	2

Source: Nigeria Centre for Disease Control (NCDC) [4]

Abbreviation: *FCT* Federal Capital Territory

## Methods

This study adopts a retrospective qualitative research design to investigate the roles and collaborations of health entities during the pandemic. The entities considered in this research include national health institutions, the WHO, and regional bodies involved in the management of COVID-19 in Nigeria.

The population of this study involves top-level directors at the NCDC, NIMR, WAHO, Lagos State COVID-19 Case Management Team, and Lagos State Primary Health Care Board (LSPHCB). Lagos State bodies were explicitly included because the state was the epicentre of the pandemic in Nigeria, recording the highest case numbers and serving as the primary hub for containment and isolation strategies.

The sample size was purposively derived to guide the interview process. Data was collected through in-depth interview sessions conducted virtually [5]. The study sample comprised seven (7) participants who are well-respected practitioners and senior directors. Interviews were audio-recorded with participant consent and lasted between 30 and 40 min. The sample size was deemed adequate as it was determined by data saturation, the point at which information on the management of the COVID-19 pandemic ceased to yield new thematic categories, satisfying qualitative research standards [6].

The primary data collection instrument was an interview guide based on the WHO’s Framework for Action for Strengthening Health Systems (2007) [7]. Ethical approval was obtained from the Covenant University Health Research Ethics Committee (CHREC/203/2023). The Consolidated Criteria for Reporting Qualitative Research (COREQ) checklist was employed to guide the comprehensive reporting of the study’s qualitative methodology and findings [8].

## Results and discussion

**Fig. 2:**
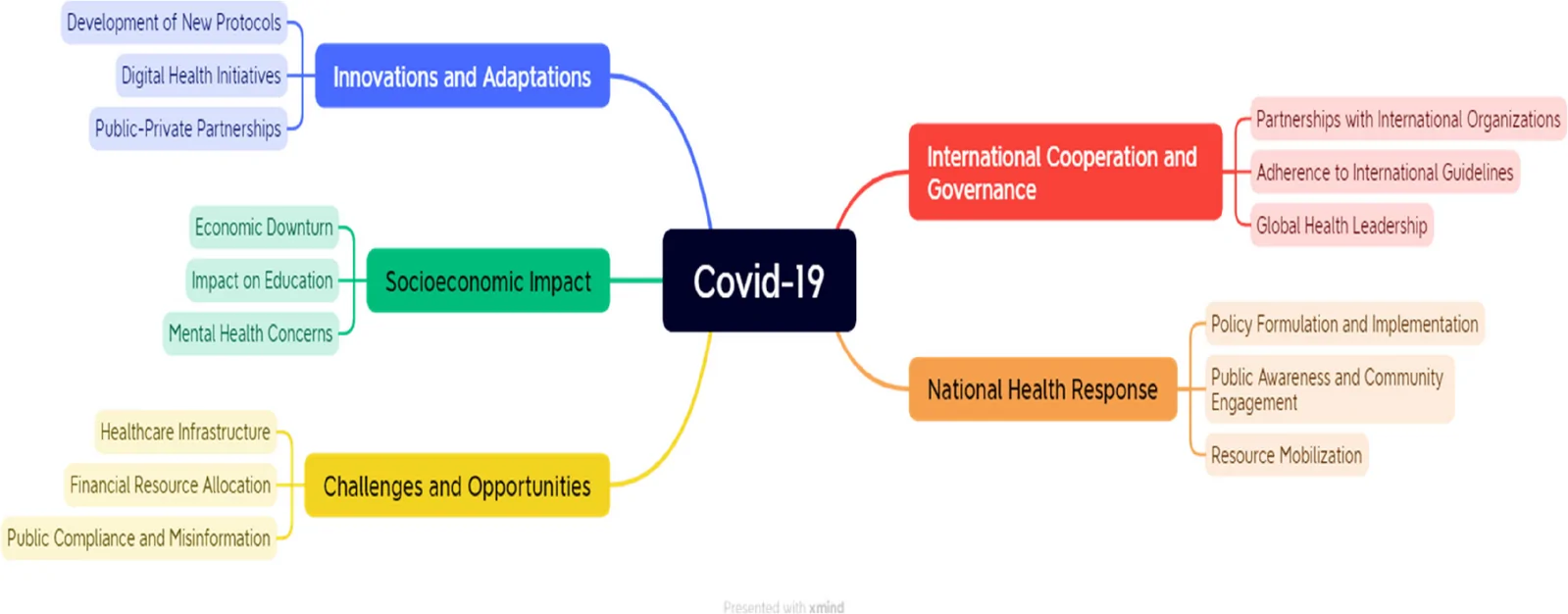
Thematic Diagram.

The overarching themes derived from the interviews are mapped out in Fig. 2. Source: Author’s Compilation. Note: The thematic diagram is a framework mapping of the associated themes covered in the study as guided by the WHO Framework for Action for Strengthening Health Systems (2007) [7].

### 1. Challenges faced during the management of the COVID-19 pandemic

#### Analytical summary

The response to the COVID-19 pandemic in Nigeria was severely tested by pre-existing infrastructural deficits, socio-economic vulnerabilities, and the widespread proliferation of misinformation. Economic lockdowns inadvertently triggered social unrest and reduced access to routine healthcare, while fake news critically undermined public compliance with safety protocols. Furthermore, as Ola et al. observed, the interactions between COVID-19 and Nigeria’s endemic public health challenges highlighted the link between infectious diseases and poverty, significantly impacting vulnerable populations [9]. Jacobs and Okeke also noted that the daily socio-economic survival needs of citizens largely invalidated the effectiveness of lockdown and curfew measures^28^.

#### Evidence from interviews

The economic and social disruptions were highlighted as primary hurdles. The Lagos COVID team respondent noted [10]: “*The lockdown slowed down the economy. This slowed the economy down to the point that it also led to social unrest*,* some believe the #ENDSARS protests were partly a result of the COVID-19 lockdown. Additionally*,* the lockdown affected healthcare delivery*,* as many people in need of medical interventions were unable to access services*”.

Compounding the infrastructural and economic challenges was the “infodemic.” The NIMR respondent emphasised [11]: “*The major challenge we had was the false information being shared on social media. A lot of misleading narratives circulated right from the beginning. There was just too much fake news out there*”. Similarly, the third NCDC respondent highlighted [12] “*inadequate healthcare infrastructure and resources*,* limited testing capabilities*,* vaccine hesitancy*,* misinformation*,* and non-compliance with preventive measures*”.

### 2. Institutional responses and collaborations to the outbreak

#### Analytical summary

**Fig. 3:**
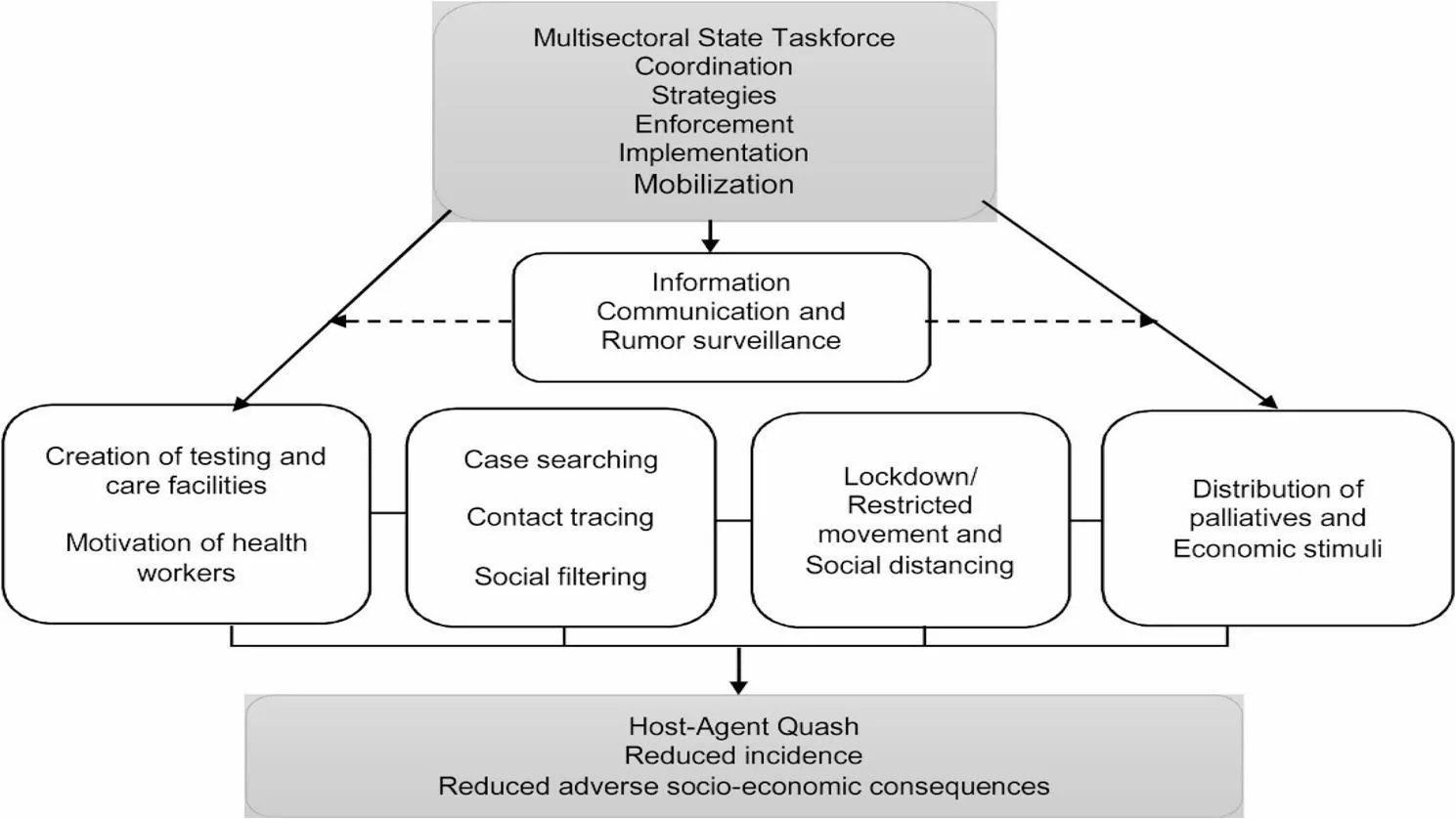
COVID-19 Response Framework [18]. Note: This framework maps out the response strategy designed to combat COVID-19 pandemic

In response to the aforementioned challenges, Nigerian health institutions adopted a highly collaborative, multi-sectoral approach. Recognizing that the pandemic could not be managed in silos, the NCDC and the PTF orchestrated a response that integrated federal, state, regional (WAHO, Africa CDC), and private sector resources (the strategy for this response is mapped in Fig. 3). This collaboration facilitated the rapid deployment of testing facilities, digital surveillance, and public health campaigns. For example, upon the confirmation of the index case, the NCDC activated the National Emergency Operations Centre to level 3, reflecting the proactive emergency operations led by National Public Health Institutes globally [13]. Collaboration extended into academia as well, with the Tertiary Education Trust Fund (TETFund) partnering with the NCDC to establish the National COVID-19 Research Consortium to conduct scientific research on the virus [2].

#### Evidence from interviews

On the role of global and regional coordination, the WAHO respondent stated [14]: “*There was strong leadership coordination from the government globally*,* and WAHO was providing leadership in the region. the formulation of the Presidential Task Force on COVID-19. was a masterstroke in interpreting policy and eliciting political will*”.

Collaboration with the private sector and international donors was critical in bridging resource gaps. The first NCDC respondent pointed out the vital role of private sector coalitions [15]: “*Public-private partnerships were established to mobilise resources*,* including funding*,* testing equipment*,* and personal protective equipment. One notable collaboration mentioned was the CA-COVID group*,* which committed significant resources to combating the pandemic*”.

At the epicentre in Lagos, coordination was equally robust. The Lagos State Primary Health Care Board respondent observed [16]: “*Nationally*,* there was a multi-sectoral team led by the Presidential Task Force*,* involving health*,* environment*,* housing*,* security*,* agriculture*,* and information. Partners*,* including UNICEF*,* WHO*,* and UNDP*,* were able to support the government*,* especially when it came to getting isolation centres*,* resources*,* and the provision of ambulances*”. 

### 3. Measures for future preparedness

#### Analytical summary

The pandemic acted as a catalyst for long-term health system strengthening. Investments in digital surveillance tools, expanded laboratory networks, and institutionalized inter-agency communication have significantly improved Nigeria’s capacity for early warning and risk reduction, directly contributing to SDG Target 3.d.

#### Evidence from interviews

Reflecting on the systemic improvements, the NIMR respondent noted [11]: “*The network of laboratories that we established is still there. We are no longer working in silos. We are able to connect and work together. There has been a clear improvement*”. The adoption of digital health tools was also a major milestone; as noted by the NIMR respondent, the NCDC’s utilisation of the Surveillance Outbreak Response Management and Analysis System (SORMAS) greatly enhanced real-time data tracking and contact tracing [17].

## Discussion of research findings

The findings of this study corroborate existing literature on the management of COVID-19 in developing nations. While Nigeria’s initial health infrastructure was fragile, the deployment of a multi-sectoral task force (the PTF) and the agility of the NCDC were critical in averting a worst-case scenario [2, 3]. Jacobs and Okeke concluded that while Nigeria’s mitigation measures contributed to a milder COVID-19 outcome compared to global trends, the response was continuously challenged by inadequate PCR testing capacity and the premature easing of lockdowns [3]. This institutional agility aligns with the global assessment by Zuber et al., who documented that National Public Health Institutes (NPHIs) worldwide were pivotal in setting testing standards, managing laboratory services, and driving human resource development during the pandemic[13]. Just as Zuber et al. observed that NPHIs took lead authority in expanding testing capacities cross-sectorally [13], the NCDC played an indispensable role in expanding testing from five to twenty-three public laboratories to manage the outbreak.

The severe impact of misinformation highlighted in this study aligns with global observations that public compliance is deeply tied to effective risk communication [18]. Crucially, the establishment of expanded laboratory networks, the integration of SORMAS for digital surveillance, and the institutionalization of public-private partnerships (such as CACOVID) demonstrate a direct fulfilment of SDG Target 3.d. By transitioning from a fragmented healthcare approach to a collaborative, digitally enabled surveillance network, Nigeria has substantially strengthened its national capacity for the early warning, risk reduction, and management of global health risks.

## Conclusion

The COVID-19 pandemic severely tested Nigeria’s national health institutions, exposing initial vulnerabilities in healthcare infrastructure, testing capacity, and public health communication. However, the crisis also triggered an unprecedented level of multi-sectoral collaboration. Through the strategic coordination of the Presidential Task Force (PTF) and the operational agility of the Nigeria Centre for Disease Control (NCDC), supported by regional bodies like WAHO and private coalitions like CACOVID, Nigeria was able to mount a robust defense. The pandemic served as a catalyst for modernizing the country’s health architecture, evidenced by the expansion of laboratory networks and the deployment of digital surveillance systems. Ultimately, these institutional adaptations represent a significant leap forward in fulfilling SDG Target 3.d, equipping Nigeria with the strengthened national capacity required for early warning, risk reduction, and the effective management of future global health emergencies.

## Supplementary Information

Supplementary Material 1.

Supplementary Material 2.

## Data Availability

The datasets generated during this study (interview transcripts) are not publicly available in order to protect the confidentiality of participants. The data are available from the corresponding author on reasonable request and subject to participant consent.
