# Supplementary material for: National health institutions and the management of COVID-19 pandemic: fulfilling the sustainable development goal three (SDG-3) in Nigeria
Source: BMC Public Health. 2026 Aug 7;26:3189. doi: 10.1186/s12889-026-28896-8 (PMC13733176; doi:10.1186/s12889-026-28896-8)
Supplement: Supplementary file 1 [file 12889_2026_28896_MOESM1_ESM.docx]

**Supplementary File S1: Interview Transcripts**

*National Health Institutions and the Management of COVID-19 Pandemic: Fulfilling the Sustainable Development Goal Three (SDG-3) in Nigeria*

Folorunso GI, Duruji MM, Chidozie FC

*Department of Political Science and International Relations, Covenant University, Nigeria*

**PREFACE**

This supplementary file contains the cleaned transcripts of seven (7) in-depth interviews conducted as part of the primary data collection for the study titled “National Health Institutions and the Management of COVID-19 Pandemic: Fulfilling the Sustainable Development Goal Three (SDG-3) in Nigeria.” The interviews were conducted virtually via teleconferencing between 2022 and 2023 and were audio-recorded with the informed consent of all participants.

Participants were purposively selected based on their direct senior-level involvement in Nigeria’s COVID-19 pandemic response. They include directors and senior officials from the Nigeria Centre for Disease Control and Prevention (NCDC), the Nigerian Institute of Medical Research (NIMR), the West Africa Health Organisation (WAHO), the Lagos State COVID-19 Case Management Team, and the Lagos State Primary Health Care Board (LSPHCB).

The interviews were structured around four thematic parts, guided by the World Health Organisation’s Framework for Strengthening Health Systems to Improve Health Outcomes (2007). The four parts are:

• Part A – Global Health Governance in Nigeria (general rating and key actors)

• Part B – COVID-19 Pandemic Management in Nigeria (effects, responses, and challenges)

• Part C – Global Governance During COVID-19 (policy guidance, intelligence and oversight, collaboration and coalition building, systems design, and accountability)

• Part D – National Initiatives in the COVID-19 Era (improvements and sustainable frameworks)

These transcripts have been lightly edited for clarity: filler words, false starts, technical interruptions, and off-topic side exchanges have been removed. The substantive content of all responses has been preserved in full. Where audio quality resulted in inaudible or indistinct passages, this is indicated within square brackets.

All participants consented verbally to participation and publication in accordance with the ethics approval granted by the Covenant University Health Research Ethics Committee (Protocol No. CHREC/203/2023). Participants are identified by name and role as consented.

**Interview 1: Prof. Rosemary Audu**

Role/Position: Director of Research; Professor of Virology

Institution: Nigerian Institute of Medical Research (NIMR); Leeds City University

Interview Date: 2023

***Part A: Global Health Governance in Nigeria***

**Interviewer:** *How would you rate global health governance in Nigeria?*

**Prof. Audu:** Top-notch. For COVID-19, particularly in Africa, the Africa CDC and the responsible committee were just on top of the game. I think it was excellent.

**Interviewer:** *Which organisations are prominent in global health governance in Nigeria?*

**Prof. Audu:** I think I have mentioned the Africa CDC. The CDC is on top of it in Nigeria. And then there is this steering committee that was providing oversight, headed by the Minister and the Director-General. That is the Presidential Task Force. Yes, those two are very germane to any research on COVID-19. The Task Force was actually behind the policies, and the NCDC was providing information, with many other institutions keeping to it to provide data.

**Interviewer:** *In what ways have the United Nations, the WHO, and other global and national stakeholders intervened in strengthening the health system in Nigeria?*

**Prof. Audu:** With respect to COVID-19, I think the WHO was actually very much in control. They provided information — even the United Nations. They were providing data up to the current time, because data and information are key in handling pandemics and outbreaks. They provided guidelines as to how to address most of these issues, and they were just on top of it. I think they did quite well. I don’t envy them; it was quite a challenge. They provided sufficient information for any country to make informed decisions, and I think most of what they provided was quite helpful for us in Nigeria.

***Part B: COVID-19 Pandemic Management in Nigeria (2020–2022)***

**Interviewer:** *In specific terms, what were the effects of the COVID-19 pandemic in Nigeria?*

**Prof. Audu:** The effect in Nigeria is similar to what we find worldwide. The closure and restriction in movement affected business. In fact, beyond the virus, the harm done was much more than the virus itself. Many people lost jobs, incomes dropped, and families were affected.

**Interviewer:** *What was the nature of the national response, and what collaborations were forged in response to the COVID-19 pandemic?*

**Prof. Audu:** The national response was quite robust. The NCDC was connecting with all the states — even though some states were not fully cooperating — coordinating activities on the ground. We were able to form networks. We also had an advisory team of which I was a member, where we gave advice to countries as test kits were approved that could be used. There was a lot of networking; it was really helpful in combating the pandemic. In terms of collaboration within Nigeria, there was a great deal of it. Let me start from my own institution. We also had the private sector coming up with their own team and supporting those in need. The support we received was immense. We had a drive-through testing facility at NIMR, and Lagos State also received support from both international organisations and the private sector. Everyone was open and willing to share resources to combat the pandemic. NIMR received funding for epidemiological surveys from institutions such as University College London, the Africa CDC, and the US CDC.

**Interviewer:** *What can you identify as the main challenges militating against the response to COVID-19 in Nigeria?*

**Prof. Audu:** The major challenge we had was the false information being shared on social media. A lot of misleading narratives circulated right from the beginning. It was like trying to win the public, who were suspicious of everything. From various parts of the world, videos were circulating supporting some dubious claims about cures and causes. There was just too much fake news out there. We were fortunate that, despite this, compliance was reasonably good. Many churches were able to hold online meetings, which have since become a permanent feature of modern life. This also opened up online virtual meetings, which have reduced costs and increased accessibility.

***Part C: Global Governance During COVID-19***

**Interviewer:** *What specific policies or strategies were formulated at the global level to control COVID-19?*

**Prof. Audu:** Strategies were being formulated even before COVID-19 came into Nigeria. These included non-pharmaceutical interventions — social distancing, wearing of masks, regular hand washing, and avoiding large gatherings. Protocols for isolation of infected individuals were also put in place early. I think they are still in place.

**Interviewer:** *Which organisations took the lead in the formulation of regional policies for intervention?*

**Prof. Audu:** At the regional level, we have the Africa CDC — that is the region we belong to — and they were majorly responsible. And then we have the West Africa Health Organisation (WAHO). Together, they have been at the forefront of these policies.

**Interviewer:** *How were these policies implemented?*

**Prof. Audu:** To a great extent, implementation was followed, particularly in urban areas. In Lagos, they enforced it — even in churches where people gathered, people were careful to keep their distance. In rural areas, I would give them about 3 out of 10 because very little was done. In the urban areas, because there was enforcement, I would score it between 6 and 7 out of 10.

**Interviewer:** *What was Nigeria’s role in this process?*

**Prof. Audu:** Nigeria complied to a great extent, though the false news that was going around really affected things. The movement restrictions were enforced — you could not travel interstate; if you tried, there were consequences. I think, in Nigeria, to a great extent, it was implemented.

**Interviewer:** *What was the level of intelligence gathering and surveillance at the national level in a bid to control COVID-19?*

**Prof. Audu:** There was a lot of it. They were having meetings and comparing data from different areas in real time. The real-time data being shared with us every day was part of the intelligence. I would rate the intelligence gathering and surveillance at 8 out of 10. It did work.

**Interviewer:** *Which organisations were charged with the responsibility of oversight?*

**Prof. Audu:** For the national response, it was the NCDC. For the global response, it was the WHO. In terms of states, Lagos was clearly ahead. Abuja was also responding, but Lagos was leading — Lagos was like a country of its own in terms of its response.

**Interviewer:** *What was the level of collaboration among Nigeria and its neighbours in the COVID-19 response?*

**Prof. Audu:** Collaboration among Nigerians was good. In terms of research and combating the pandemic, even those that travelled through airports were subjected to surveillance to ensure containment. The borders — both land and air — were being monitored.

**Interviewer:** *How were local and international NGOs and civil society groups involved in the COVID-19 response?*

**Prof. Audu:** They played a good role. They were quite active — out there in their different ways, trying to control the spread and responding to the pandemic. For example, the drive-through testing facility at NIMR was established through such partnerships. We have a long list of those who partnered with us in our published response reports.

**Interviewer:** *Are there any known coalitions or public-private partnerships that were formed to control and contain COVID-19 at the national level?*

**Prof. Audu:** At the national level, yes, I believe so. There is a group that now meets every two weeks, beyond COVID-19, to discuss other issues of concern. It has been continued for preparedness.

**Interviewer:** *How were human and material resources pooled at the national level?*

**Prof. Audu:** Human resources — people volunteered. In Nigeria, we have a FELTP programme that has a coordinated group of trained personnel. They were just mobilised and deployed to respond. Similarly for material resources — they were provided and distributed, though the distribution might not have been perfectly equitable across all areas.

**Interviewer:** *What specific national initiative helped Nigeria contain COVID-19?*

**Prof. Audu:** The SORMAS software was a significant one. I think the NCDC really made good use of it. That was a specific and practical initiative that helped in tracking, contact tracing, and data management.

**Interviewer:** *Was there any national organisation charged with the responsibility of managing resources in response to COVID-19?*

**Prof. Audu:** Honestly, I don’t know. I cannot confirm whether there was a specific body formally charged with that responsibility. I can only speak for my institution.

**Interviewer:** *How were those funds managed in your opinion?*

**Prof. Audu:** I can only speak for my institution. The funds given to us for COVID-19 intervention were used for COVID-19 intervention. We had to give detailed reports, publish evidence, and show how we spent them. We had auditors who came to check our reports and accounts.

**Interviewer:** *What is your assessment of the national role in the response to COVID-19?*

**Prof. Audu:** I think we did well. It was satisfactory. The national role was commendable.

***Part D: National Initiatives in the COVID-19 Era***

**Interviewer:** *Have there been any noticeable improvements in national thinking and governance since COVID-19?*

**Prof. Audu:** Yes. The network of laboratories that we established is still there. From time to time, people who have concerns can post and get contributions from every part of the country. We are no longer working in silos. We are able to connect and work together. There has been a clear improvement.

**Interviewer:** *What are the global actors’ contributions to new national initiatives?*

**Prof. Audu:** At the global level, there are encouraging efforts around vaccine development. Countries are now being encouraged to look inward and achieve self-reliance. Because of the lockdown, we were unable to import things as before. Now we are looking inside, developing local capacity, and I think we are making great improvement in that aspect.

**Interviewer:** *How can Nigeria develop a sustainable national framework for health governance?*

*[Note: The audio recording ended at this point; the interviewee’s response to this final question was not captured in the transcript.]*

**Interview 2: Dr. Segun Adeoye**

Role/Position: Coordinator, Regional Disease Surveillance Systems (REDISSE) Project; Acting General Coordinator

Institution: West Africa Health Organisation (WAHO)

Interview Date: 2023

***Part A: Global Health Governance in Nigeria***

**Interviewer:** *How would you rate global health governance in Nigeria?*

**Dr. Adeoye:** Well, global health governance in Nigeria is generally pretty straightforward. We have two layers to consider. We have the general layer provided by the World Health Organisation through its frameworks and guidelines, and then we have the adaptation layer at the country level. The adaptation of global health governance in Nigeria has faced challenges particularly around resource mobilisation and human capacity. The will to contribute to implementation is usually not fully there. I would say the adaptation process warrants a rating of around 5 or 6 out of 10.

**Interviewer:** *Which organisations are prominent in global health governance in Nigeria?*

**Dr. Adeoye:** At the regional level, we are talking about the West Africa Health Organisation (WAHO), because when we are doing adaptation, it involves every country in West Africa. Then we have bilateral organisations like USAID, which is prominent in health security, HIV, and many other health interventions in Nigeria. They support the government in adaptation. Then we also have the multilateral organisations such as the World Bank, UNICEF, and others.

**Interviewer:** *In what ways have the United Nations, the WHO, and other stakeholders intervened in strengthening the health system in Nigeria?*

**Dr. Adeoye:** All those international organisations like the UN — the WHO especially — work mainly on policy formation: global policy and global direction. And also occasional interventions. I think the WHO has been doing really well. You could see this in the advent of COVID-19, when they were able to periodically release clear guidelines and updates, and to guide countries on the best approaches to take.

***Part B: COVID-19 Pandemic Management in Nigeria (2020–2022)***

**Interviewer:** *In specific terms, what were the effects of COVID-19 in Nigeria?*

**Dr. Adeoye:** For me, the most important specific impact is that it affected the economy significantly. It threw a lot of people below the poverty line. And we all know that when the financial space is disrupted, health suffers considerably because it is one of the first casualties of economic hardship.

**Interviewer:** *What was the nature of the national response, and what collaborations were forged?*

**Dr. Adeoye:** I think the national response was very, very solid. First, there was strong leadership coordination from the government globally, and WAHO was providing leadership in the region. But the most important thing was Nigeria’s ability to respond effectively through the NCDC. They provided strong coordination. And then, to show political will, there was the formulation of the Presidential Task Force on COVID-19, which I think was a masterstroke in interpreting policy and eliciting political will. However, I think we took our eyes off the ball when the borders were opened and the perception developed among some that they were no longer susceptible to the effects of the virus. We then started hearing issues about mismanagement of COVID-19 funds. But prior to that, there was a strong, effective, and robust response system in terms of demand creation, in terms of supply, and in terms of coordination.

**Interviewer:** *What were the main challenges militating against the response to COVID-19 in Nigeria?*

**Dr. Adeoye:** The major challenge, for me, was the community awareness and the issue of misinformation. People did not fully understand COVID-19, and this led to non-compliance with health measures. The lack of community education about COVID-19 severely impacted the response efforts, as misinformation and rumours led to non-compliance with the measures implemented.

***Part C: Global Governance During COVID-19***

**Interviewer:** *What specific policies or strategies were formulated at the global level to control COVID-19?*

**Dr. Adeoye:** We have been developing global health security policies and guidelines. We also had the COVID-19 sports guidelines — guidelines for conducting sporting events during the pandemic — as well as other regional bodies developing their own frameworks. Policies included stockpiling of commodities and response strategies, which were adapted for domestic use.

**Interviewer:** *Which organisations took the lead in the formulation of such regional policies?*

**Dr. Adeoye:** At the West African level, it was WAHO. We lobbied health ministries to adopt guidelines, which were then translated into domestic implementation by the respective countries. One who formulates a policy and guides its implementation must also ensure it is approved through proper channels, and that is what WAHO did.

**Interviewer:** *How were these policies implemented?*

**Dr. Adeoye:** The first level of implementation is to get the buy-in of the assemblies of health ministers. When there is an approval, the ministers translate it to domestic implementation. Early in the pandemic, health ministers took COVID-19 very seriously, leading to widespread adoption of measures across various countries. WAHO also conducted national training programmes to educate and raise awareness among healthcare workers.

**Interviewer:** *What was Nigeria’s role in this process?*

**Dr. Adeoye:** Nigeria’s role was firstly to receive support in terms of guideline documentation and policy direction in health, and then to ensure they do what they can to implement those guidelines. Nigeria is a member of the global village, so whatever policy was given by the global community, Nigeria was expected to follow, and it did so to a commendable extent. Nigeria also developed some of its own policies that complemented the global ones.

**Interviewer:** *What was the level of intelligence gathering and surveillance at the national level?*

**Dr. Adeoye:** I think it was very significant. I’m sure you saw the daily reporting. For the first time in the history of Nigeria following the Ebola outbreak, we had a situation where we did not report more than one case of Ebola — and then we had a lot of updates from the Ministry. I think Nigeria did well in that area. The real-time data reporting was commendable.

**Interviewer:** *Which organisation was charged with the responsibility of oversight?*

**Dr. Adeoye:** In terms of oversight, it was a collective effort. There was a COVID-19 Management Coordinating Committee that included all stakeholders — World Bank, USAID, WHO, and others. WAHO also played a supporting role in developing cross-border strategies and conducting training exercises, facilitating the exchange of information between countries regarding disease notifications.

**Interviewer:** *What was the level of collaboration among Nigeria and its neighbours in the COVID-19 response?*

**Dr. Adeoye:** It was very important — very, very important. Through WAHO, we supported Nigeria and other West African countries to develop a cross-border strategy. We also did cross-border exercises. It was not fully established at first, but now we can say that all the countries are on board.

**Interviewer:** *How were local and international NGOs and civil society groups involved in the COVID-19 response?*

**Dr. Adeoye:** They were involved a great deal. Many did community mobilisation, health risk management, distribution of test kits, distribution of commodities such as hand sanitisers and personal protective equipment. They also supported vaccination efforts.

**Interviewer:** *Are there any known coalitions or public-private partnerships formed to control COVID-19 nationally?*

**Dr. Adeoye:** Coalitions evolved according to the situation. I think we had enough coalitions for the COVID-19 response. The key one at the national level was the Presidential Task Force on COVID-19, which was charged with the responsibility of pooling resources and providing overall coordination. The establishment of NCDC as a national public health institution dedicated to epidemic management was also a critical national initiative.

**Interviewer:** *Was any national organisation charged with the responsibility of managing resources in response to COVID-19?*

**Dr. Adeoye:** I don’t think it was formally charged to one specific organisation. All organisations took charge of their own funding support to the country in different frameworks. For example, the World Bank would create a system under its own framework, other partners under theirs. The funds were managed well until the borders were re-opened — that is when coordination began to break down.

**Interviewer:** *How did Nigeria contribute to the overall global response to COVID-19?*

**Dr. Adeoye:** I think we did pretty well. We were reporting a lower case fatality ratio relative to the region. Although our case numbers were among the highest in West Africa, our response was effective. On a scale of 1 to 10, I would give the national response a 7.

**Interviewer:** *What were the challenges militating against national response?*

**Dr. Adeoye:** Community awareness and preparedness, and the availability of expertise for solving response challenges were the main issues.

***Part D: National Initiatives in the COVID-19 Era***

**Interviewer:** *Have there been any noticeable improvements in national thinking and governance since COVID-19?*

**Dr. Adeoye:** I think Nigeria has improved significantly. COVID-19 has, in a way, created awareness, political will, and driven the quest for health system strengthening. It led to a level of investment and attention to public health institutions that was not there before. Global actors have also contributed significantly, because they are often part of the process for developing those initiatives. It is a multidisciplinary and multi-sectoral approach, and that is what has been driving improvement.

**Interviewer:** *How can Nigeria develop a sustainable national framework for health governance?*

**Dr. Adeoye:** First, we need political will for the availability of funding. Second, we need more skilled health workers. We already have the guideline and policy frameworks in place. What we need is a policy that ensures financial commitment, stakeholder buy-in, and human resource training for consistent and accountable implementation.

**Interview 3: Dr. Bowale Abimbola**

Role/Position: Medical Director, Infectious Disease Hospital (Mainland Hospital, Yaba); Member, Lagos State COVID-19 Case Management Team

Institution: Lagos State Government / Mainland Hospital, Lagos

Interview Date: 2023

***Part A: Global Health Governance in Nigeria***

**Interviewer:** *How would you rate global health governance in Nigeria?*

**Dr. Abimbola:** Very good. It has been excellent because Nigeria is part of the globalised world, and whatever affects any country will also affect us in Nigeria. We are part of many health initiatives worldwide, and Nigeria’s response to any global health concern is therefore integrated with global mechanisms. On a scale of 1 to 10, I would say 6.

**Interviewer:** *Which organisations are prominent in global health governance in Nigeria?*

**Dr. Abimbola:** The WHO is one of them — very, very key. The Africa CDC is also very key. Then the West African countries also have their own body — the West Africa Health Organisation (WAHO). There are also other global institutions involved in specific aspects of health, like the Global Fund for tuberculosis and other diseases, and the World Bank, which was especially involved with COVID-19.

**Interviewer:** *In what ways have the United Nations, the WHO, and other stakeholders intervened in strengthening the health system in Nigeria?*

**Dr. Abimbola:** Training is one of the key ways. The World Bank is involved, especially with COVID-19. They provide training, they provide materials for research, they provide consumables and other necessary items for interventions such as setting up laboratories, through grants provided to their agencies working in Nigeria.

***Part B: COVID-19 Pandemic Management in Nigeria (2020–2022)***

**Interviewer:** *In specific terms, what were the effects of COVID-19 in Nigeria?*

**Dr. Abimbola:** We had the economic impact of COVID-19. The lockdown slowed down the economy because people could not run their businesses. This slowed the economy down to the point that it also led to social unrest — some believe the #ENDSARS protests were partly a result of the COVID-19 lockdown. The inability to conduct business and earn income caused widespread restlessness. Additionally, the lockdown affected healthcare delivery, as many people in need of medical interventions were unable to access services. People also became unable to afford healthcare — even those who previously could pay out-of-pocket expenses found themselves unable to do so because of lost income.

**Interviewer:** *What was the nature of the national response, and what collaborations were forged?*

**Dr. Abimbola:** The national response was actually very quick and well-coordinated. The NCDC is in charge of such responses in Nigeria, and they coordinated the response across all the states. The federal government was also involved in terms of setting up the Presidential Task Force on COVID-19 to oversee all the responses in all the states. The World Bank actively provided funding and training. The collaboration between the African CDC, WAHO, the Nigerian CDC, and State Ministries of Health was outstanding.

**Interviewer:** *What can you identify as the main challenges militating against the response to COVID-19 in Nigeria?*

**Dr. Abimbola:** Ignorance would be one of them — ignorance on the part of the populace, and the fact that some of them did not believe that COVID actually existed. This ignorance bred so many problems, including resistance to vaccines and non-compliance with health protocols. Misinformation was also one of the main challenges. Each state’s incident command system included a social mobilisation arm tasked with enlightening the community. However, widespread ignorance and misinformation, especially on social media, exacerbated the situation.

***Part C: Global Governance During COVID-19***

**Interviewer:** *What specific policies or strategies were formulated at the global level to control COVID-19?*

**Dr. Abimbola:** The major one was the declaration of COVID-19 as a Public Health Emergency of International Concern (PHEIC) by the WHO. That declaration at the global organisational level helped unlock funds and mobilise resources internationally.

**Interviewer:** *Which organisations took the lead in the formulation of regional policies?*

**Dr. Abimbola:** At the global level, the WHO. At the African level, the Africa CDC was also very active. At the West African level, we have WAHO. And then in Nigeria, the NCDC and the Lagos State incident command system through the State Ministry of Health.

**Interviewer:** *How were these policies implemented?*

**Dr. Abimbola:** Once the PHEIC was declared, member states of the WHO were also enabled and empowered to implement measures to contain COVID-19. One of the measures implemented almost globally was the lockdown, which restricted movement and gatherings.

**Interviewer:** *What was Nigeria’s role in this process?*

**Dr. Abimbola:** The NCDC was actually there to ensure control of any outbreak of infectious diseases in the country. The NCDC was very active in terms of training, providing logistics support for states to combat COVID-19. The Presidency also set up the Presidential Task Force, which coordinated activities related to COVID-19 at the national level.

**Interviewer:** *What was the level of intelligence gathering and surveillance at the national level?*

**Dr. Abimbola:** It was also very high. The NCDC was in charge, and the Lagos State Government also had its own team doing all the surveillance work. Surveillance is a very strong arm of the incident command system. Regular meetings were held to compare real-time data from various areas, which facilitated intelligence gathering.

**Interviewer:** *Which organisations were charged with the responsibility of oversight?*

**Dr. Abimbola:** For the national response, it was the NCDC. For the state level, it was the State Ministry of Health. For the global response, the WHO oversaw the international response until COVID-19 became a thing of the past.

**Interviewer:** *What was the level of collaboration among Nigeria and its neighbours in the COVID-19 response?*

**Dr. Abimbola:** It was excellent. The West Africa Health Organisation (WAHO), as the coordinating body for health in West Africa, ensured support to Nigeria and other West African countries in developing cross-border strategies.

**Interviewer:** *How were local and international NGOs and civil society groups involved in the COVID-19 response?*

**Dr. Abimbola:** Quite a number of them were very active. The CA-COVID coalition — the Coalition Against COVID-19 — was heavily involved in the management and control of COVID-19 in Nigeria. It was the private sector-led coalition that I am most aware of.

**Interviewer:** *Was any national organisation charged with the responsibility of managing resources in response to COVID-19?*

**Dr. Abimbola:** That question would be better addressed by those in the Ministry — either the Permanent Secretary or the Commissioner for Health. I may not be able to answer that question directly from my position.

**Interviewer:** *How did Nigeria respond to COVID-19 and what is your assessment of the national role?*

**Dr. Abimbola:** It was good. It was very excellent. At the beginning of the epidemic, the position of Nigeria in terms of infection and death rates was very alarming. But because of the fact that the NCDC and the Presidential Task Force were well-coordinated, they were able to bring it under control. I would rate it 9 out of 10.

**Interviewer:** *What were the challenges militating against the national response?*

**Dr. Abimbola:** Ignorance, misinformation, and limitations in testing capacity. We had some limitation in the number of testing laboratories available. These are the key challenges I would highlight.

***Part D: National Initiatives in the COVID-19 Era***

**Interviewer:** *Have there been any noticeable improvements in national thinking and governance since COVID-19, including in intelligence and oversight, systems design, and collaboration?*

**Dr. Abimbola:** Definitely. After Ebola in 2014, the country has been very proactive in ensuring that things are done differently. In terms of intelligence and surveillance, yes, there has been definite improvement. In terms of systems design and collaboration, there have also been improvements. There are several checks and balances, financial regulations, and policies that we as a nation and as civil servants have to adhere to. I think these checkmates have helped ensure accountability.

**Interviewer:** *What are the global actors’ contributions to these new national initiatives?*

**Dr. Abimbola:** We work hand in hand with the agencies I mentioned earlier — the Africa CDC, the West Africa Health Authority (WAHO), the Nigerian NCDC, and the State Ministries of Health. There is very robust collaboration among all of us.

**Interviewer:** *How can Nigeria develop a sustainable national framework for health governance?*

**Dr. Abimbola:** Policy implementation is key, along with adequate funding. And of course, before you formulate a policy, you must get all the stakeholders’ buy-in. Once you have all stakeholders’ buy-in, and policy implementation is followed through with adequate funding, you can then build a truly sustainable national framework for health governance.

**Interview 4: Dr. Dapo Asiyanbi**

Role/Position: Senior Official

Institution: Lagos State Primary Health Care Board (LSPHCB)

Interview Date: 2023

***Part A: Global Health Governance in Nigeria***

**Interviewer:** *How would you rate global health governance in Nigeria?*

**Dr. Asiyanbi:** I will give it a 7 out of 10.

**Interviewer:** *Which organisations are prominent in global health governance in Nigeria?*

**Dr. Asiyanbi:** Locally, we have governmental organisations: the Presidential Task Force (PTF), which is the coordinating arm; the Federal Ministry of Health; and governance at the state level. At the implementation level, you have all the partners that have supported the government: organisations such as UNICEF, WHO, UNFPA, and other UN agencies. Then you have other non-governmental partners, international organisations, private donors, and philanthropic foundations.

**Interviewer:** *In what ways have the United Nations, the WHO, and other stakeholders intervened in strengthening the health system in Nigeria?*

**Dr. Asiyanbi:** Because of their mandates, most of them come in and use their existing disease response infrastructure to support the health system. They have focused areas and they provide resources, including capacity building, in those areas. Those resources and capacity can also be used for COVID-19. For example, we had a surveillance network that had been built up using the polio eradication programme. It was easy for us to use those community surveillance teams to lead the COVID-19 response, because we had to resort to house-to-house reporting and notification. We also had people working as TB surveillance officers and community mobilisers for different programmes, so it was easy to incorporate them into the COVID-19 response.

***Part B: COVID-19 Pandemic Management in Nigeria (2020–2022)***

**Interviewer:** *In specific terms, what were the effects of COVID-19 in Nigeria?*

**Dr. Asiyanbi:** There was a significant socioeconomic effect. Due to restrictions in movement, goods and services could not move, leading to an economic lockdown. This made poor people very vulnerable, as some of them could not afford basic necessities. Fear was also rampant, as upper respiratory tract infections — common symptoms of COVID-19 — led to immediate suspicion of the virus, complicating care provision even among health workers. Healthcare became more expensive, and out-of-pocket expenses increased significantly.

**Interviewer:** *What was the nature of the national response, and what collaborations were forged?*

**Dr. Asiyanbi:** Nationally, there was a multi-sectoral team led by the Presidential Task Force, involving health, environment, housing, security, agriculture, and information. Partners — including UNICEF, WHO, and UNDP — were able to support the government, especially when it came to getting isolation centres, resources, and the provision of ambulances. Non-governmental partners, local organisations, the private sector, and private donors also contributed to community responses, including in the area of diagnostics.

**Interviewer:** *What were the main challenges militating against the COVID-19 response in Nigeria?*

**Dr. Asiyanbi:** I would say two main things. First, the inadequate recognition that the best way to strengthen the pandemic response is to strengthen the primary healthcare system at the community level. Not much was done to strengthen this level, even though the majority of people had their interactions with the health system at the primary care level. Second, border movement and trade restrictions were a significant pain point, because nobody wanted anyone to bring the virus from outside. The management of cross-border transmission posed real challenges.

***Part C: Global Governance During COVID-19***

**Interviewer:** *What specific policies or strategies were formulated at the global level to control COVID-19?*

**Dr. Asiyanbi:** The key policies were around reducing contact: regular hand washing, mandatory masking, and the isolation of any infected person as quickly as possible. Policies were also formulated around quarantine and isolation in treatment centres, vaccination rollout, and travel restrictions.

**Interviewer:** *Which organisations took the lead in the formulation of such policies?*

**Dr. Asiyanbi:** In Nigeria, it was the Presidential Task Force and the NCDC that led policy formulation and implementation. Regionally, we had the West Africa Health Organisation (WAHO) and the Africa CDC.

**Interviewer:** *What was Nigeria’s role in the global response?*

**Dr. Asiyanbi:** Nigeria is a member of the global community. Whatever policy direction was given by the global village, Nigeria followed — as most countries did. Nigeria also developed some of its own complementary policies that were adapted to local conditions.

**Interviewer:** *How were local and international NGOs involved in the COVID-19 response?*

**Dr. Asiyanbi:** They gave resources, they gave their time, and they worked with governmental organisations to manage COVID-19 outcomes: contact tracing, treatment, isolation, and eventually vaccination.

**Interviewer:** *How did Nigeria contribute to the overall global response to COVID-19?*

**Dr. Asiyanbi:** Nigeria contributed by controlling the pandemic as much as it could within its borders. The country set up proper means of integration for surveillance using early warning systems, which contributed to the global picture of the pandemic.

**Interviewer:** *What is your assessment of the national role in the response to COVID-19?*

**Dr. Asiyanbi:** Maybe a 7 out of 10, based on the existing parameters.

**Interviewer:** *What were the challenges militating against national response?*

**Dr. Asiyanbi:** Lack of resources and inadequate community awareness about what to expect going forward.

***Part D: National Initiatives in the COVID-19 Era***

**Interviewer:** *Have there been any noticeable improvements in national thinking and governance since COVID-19?*

**Dr. Asiyanbi:** In the area of intelligence and oversight, yes. The structures are still there. There has been improvement in diagnostic capacity, and this has benefited the management of other diseases in the Nigerian spectrum as well.

**Interviewer:** *How can Nigeria develop a sustainable national framework for health governance?*

**Dr. Asiyanbi:** By showing that at the local level — the community and primary healthcare level — there is governance using community ownership and locally mobilised funds to manage health challenges. Community ownership is the foundation of a sustainable national health governance framework.

**Interview 5: Dr. Chinwe Ochu**

Role/Position: Director, Planning, Research and Statistics

Institution: Nigeria Centre for Disease Control and Prevention (NCDC)

Interview Date: 2023

***Part A: Global Health Governance in Nigeria***

**Interviewer:** *How would you rank Nigeria’s global health governance?*

**Dr. Ochu:** In recent years, particularly in reaction to the COVID-19 epidemic, global health governance in Nigeria has significantly improved. The government has demonstrated a strong commitment to working in partnership with international organisations and stakeholders to create and carry out health policies and plans. However, there are still some areas that need greater collaboration and development.

**Interviewer:** *Which organisations are prominent in global health governance in Nigeria?*

**Dr. Ochu:** In Nigeria, the World Health Organisation (WHO) is crucial to the governance of global health. They offer technical help, direction, and coordination in areas such as capacity building, surveillance, and policy formulation. The global health governance initiatives in Nigeria also receive active support from other organisations including the United Nations (UN) and numerous international donor agencies.

**Interviewer:** *What steps have the UN, WHO, and other international and national stakeholders taken to improve Nigeria’s healthcare system?*

**Dr. Ochu:** The WHO and the UN have been crucial in assisting Nigeria’s healthcare system. They have contributed technical know-how, financial resources, and capacity building programmes to improve the healthcare system, disease surveillance, laboratory systems, and the overall response to public health emergencies. The financial and resource mobilisation efforts of other parties, including international NGOs and donor organisations, have significantly aided Nigeria’s health system.

***Part B: COVID-19 Pandemic Management in Nigeria (2020–2022)***

**Interviewer:** *What were the pandemic’s specific impacts in Nigeria?*

**Dr. Ochu:** Nigeria was significantly impacted by the COVID-19 pandemic. A sizable number of confirmed cases, hospitalisations, and unfortunately fatalities occurred. Our healthcare system was strained by the pandemic, and there were setbacks in the economy, social interactions, and education, among other areas. Additionally, it brought attention to population vulnerabilities and pre-existing health disparities.

**Interviewer:** *What was the nature of the national response to the COVID-19 epidemic, and what partnerships were established?*

**Dr. Ochu:** The government provided strong leadership and there was active collaboration between a number of partners in the national response to the COVID-19 epidemic. The Federal Ministry of Health and the NCDC worked together to lead the response operations. We coordinated surveillance, testing, contact tracing, treatment, and public health messaging in close collaboration with state governments, public and private healthcare institutions, foreign partners, local NGOs, and community leaders. The CA-COVID coalition was also conceived by the private sector, allowing for private sector-driven financial support throughout the pandemic.

**Interviewer:** *What significant obstacles affected Nigeria’s reaction to the COVID-19 pandemic?*

**Dr. Ochu:** The Nigerian reaction to the COVID-19 outbreak was hampered by a number of issues. These included inadequate testing capabilities, inaccurate information and rumours, disregard for preventive actions, and non-compliance with public health recommendations. Additionally, it was difficult to reach isolated areas and provide equal access to testing and medical care. Our response efforts were further hampered by vaccine reluctance and the emergence of mutant strains.

***Part C: Global Governance During COVID-19***

**Interviewer:** *What concrete regulations or plans of action were developed on a global scale to manage COVID-19?*

**Dr. Ochu:** To regulate COVID-19, a number of policies and initiatives were developed on a global scale. Among these were protocols for testing, contact tracing, isolation and quarantine, treatment, and public health messaging. Technical advice and recommendations were provided by the WHO in cooperation with other international organisations, supported by research and industry standards.

**Interviewer:** *Which organisation or organisations took the initiative in developing regional intervention policies?*

**Dr. Ochu:** The World Health Organisation played a key role in developing regional policies and COVID-19 measures. They collaborated closely with regional health organisations like the West Africa Health Organisation (WAHO) and the Africa Centres for Disease Control and Prevention (Africa CDC) to create coordinated strategies and response plans catered to the unique requirements of African nations like Nigeria.

**Interviewer:** *What role did Nigeria play in the implementation of these policies and strategies?*

**Dr. Ochu:** Coordination between national and subnational levels as well as adaptability to the local context were required for the execution of global policies and plans. Nigeria actively collaborated with the WHO and other international partners through the NCDC and the Federal Ministry of Health to align our response activities with international standards. We took part in initiatives to build capacity, share information, and provide frequent updates on the epidemiological situation, all of which helped to hone and strengthen international policies.

**Interviewer:** *How extensive was national intelligence gathering and surveillance in an effort to contain COVID-19?*

**Dr. Ochu:** During the COVID-19 pandemic, the NCDC, which is responsible for disease control and surveillance in Nigeria, carried out intensive intelligence collection and surveillance operations. We developed a network of laboratories for testing and genomic surveillance, enhanced our surveillance systems, and deployed real-time data collection and processing. As a result, we were able to monitor the virus’s spread, identify new variants, and plan our responses and containment measures with informed knowledge.

**Interviewer:** *What government agency or institution was tasked with maintaining oversight over both the international and domestic responses?*

**Dr. Ochu:** The World Health Organisation was in charge of overseeing the global response. For oversight, coordination, and monitoring of the national response at the federal level, the NCDC and the Federal Ministry of Health were in charge. Cooperation with state governments, health agencies, and other relevant parties was required to guarantee that response measures were implemented successfully.

**Interviewer:** *What was the extent of cooperation between Nigeria and its neighbours in the COVID-19 response?*

**Dr. Ochu:** The COVID-19 response required collaboration between Nigeria and its neighbours. The West African Health Organisation (WAHO) aided ECOWAS member states in regional coordination and information sharing. We worked together to stop the virus from spreading in the region through frequent meetings, the exchange of best practices, and coordinated actions. The synchronisation of policy, border security measures, and surveillance operations was made possible by this collaboration.

**Interviewer:** *How did national and international NGOs and civil society organisations participate in the COVID-19 response?*

**Dr. Ochu:** The COVID-19 response in Nigeria was greatly aided by national and international NGOs as well as civil society organisations. They offered assistance in various areas, including promoting vulnerable groups, risk communication, distributing aid, and engaging the community. Reaching out to rural areas, countering myths, and encouraging adherence to preventive measures were all made possible through their cooperation. The NCDC worked closely with these organisations, utilising their knowledge and networks.

**Interviewer:** *Were any public-private partnerships or non-coalition organisations established to manage and contain COVID-19 on a nationwide scale?*

**Dr. Ochu:** In order to control and contain COVID-19 at the national level, numerous non-coalition and public-private partnerships were established. These alliances involved working with for-profit businesses, charitable foundations, and private healthcare providers. They supplied materials, funding, and technical know-how to build isolation and treatment facilities, fund public health campaigns, and increase testing capacity. The CA-COVID alliance, which was active during the epidemic, is a significant example. These collaborations were essential to boosting government response efforts and guaranteeing a planned strategy.

**Interviewer:** *How were national human and material resources combined in the COVID-19 response?*

**Dr. Ochu:** A crucial component of the national response to COVID-19 was the pooling of human and material resources. The Federal Ministry of Health and the NCDC worked together to organise the mobilisation of medical personnel, including volunteers and staff members from various healthcare facilities. Resources such as personal protective equipment (PPE), testing kits, and medical supplies were additionally procured and distributed across the nation, with assistance from international partners and regional manufacturers.

**Interviewer:** *What unique national measure helped the nation contain COVID-19?*

**Dr. Ochu:** The formation of the Presidential Task Force on COVID-19 was one particular national initiative that contributed significantly to the containment of COVID-19. The Secretary to the Government of the Federation served as the chair of this task force, which oversaw response activities and provided high-level coordination and policy direction. It facilitated regular briefings, risk analyses, and the implementation of preventive measures, all of which helped keep the infection under control.

**Interviewer:** *Was there a national agency tasked with overseeing the allocation of resources in response to COVID-19?*

**Dr. Ochu:** Yes, the NCDC and the Presidential Task Force on COVID-19 worked together to manage resources in response to COVID-19. This entailed coordinating the allocation of resources among different states and healthcare facilities, as well as procuring medical supplies. The NCDC was essential in monitoring these resources and establishing accountability for their use.

**Interviewer:** *How, in your judgement, was the management of those funds?*

**Dr. Ochu:** In my judgement, an open and accountable approach was used to manage the funds. In conjunction with the relevant government authorities, the Presidential Task Force on COVID-19 adopted stringent financial management policies, including auditing and monitoring systems. Regular reports and updates on resource allocation and utilisation were provided to promote transparency and accountability.

**Interviewer:** *What role did Nigeria play in the global response to COVID-19?*

**Dr. Ochu:** Nigeria significantly impacted the overall worldwide response to COVID-19. Participating actively in international forums, we shared our knowledge, best practices, and lessons learned. Through the dispatch of medical staff, the exchange of testing and surveillance know-how, and participation in regional campaigns for the distribution of vaccines, we also supported other African nations. Nigeria’s experience and lessons have helped the world better understand the pandemic and develop effective response methods.

**Interviewer:** *What evaluation would you make of the national roles in the COVID-19 response?*

**Dr. Ochu:** In general, I think Nigeria made a strong and proactive contribution to the COVID-19 response. The government, in partnership with the NCDC and other stakeholders, exhibited leadership, resilience, and adaptation in the face of a complex and fast-changing crisis. We created and implemented solutions specifically tailored to our local environment, participated in international partnerships, and mobilised resources.

**Interviewer:** *What obstacles, if any, prevented a stronger national response?*

**Dr. Ochu:** The national response to COVID-19 encountered a number of difficulties. These included inadequate healthcare infrastructure and resources, a lack of testing capability, reluctance to receive vaccinations, misinformation, and disregard for preventive actions. Scaling up the response and ensuring equitable access to healthcare were also made difficult by a lack of funding and resources. Our response efforts were also continually hampered by the introduction of new variants and the requirement for continued public awareness and behaviour change.

***Part D: National Initiatives in the COVID-19 Era***

**Interviewer:** *Since the COVID-19 epidemic broke out, have there been any appreciable advancements in national thinking and governance in the areas of policy guidance, intelligence and oversight, system design, collaboration and coalition building, and accountability?*

**Dr. Ochu:** Yes, there are observable gains in many aspects of national thinking and governance as a result of the COVID-19 pandemic. The epidemic necessitated a review and modification of current regulations as well as the creation of new guidelines to address emerging problems. Through investments in surveillance technology, data collection, and analysis for evidence-based decision-making, intelligence and oversight capacities were boosted. With the creation of specialised isolation and treatment facilities, the growth of laboratory networks, and the improvement of healthcare infrastructure, system design was enhanced. The importance of cooperation and coalition building resulted in deeper alliances between international stakeholders, private sector companies, and government agencies. Additionally, accountability systems were strengthened to guarantee open resource allocation and efficient response activity tracking.

**Interviewer:** *What contributions, if any, do international players provide to these new national initiatives?*

**Dr. Ochu:** The World Health Organisation and other international partners have made significant contributions to and support for these new national efforts. They have contributed technical know-how, financial resources, capacity building initiatives, and forums for knowledge exchange. The sharing of best practices, lessons learned, and opportunities for international cooperation have also been facilitated by global actors. Their contributions have improved the viability and effectiveness of national responses to the COVID-19 pandemic.

**Interviewer:** *How can Nigeria create a national framework for health governance that is sustainable?*

**Dr. Ochu:** A multifaceted strategy is needed to create a sustainable national framework for health governance. First and foremost, there is a requirement for ongoing investments in healthcare infrastructure, which includes enhancing healthcare systems, extending laboratory networks, and improving surveillance and data management capabilities. Policy frameworks should be robust, founded on evidence, and adaptable to emerging health challenges. To guarantee a coordinated and comprehensive approach to health governance, collaboration and coordination should be encouraged among government agencies, private sector businesses, civil society organisations, and international partners. Adequate financing, resource mobilisation, and accountability systems are crucial for sustained implementation. Lastly, placing an emphasis on health education, awareness, and community participation will enable people and communities to take charge of their health and contribute to long-term health governance.

**Interview 6: Dr. John Oladejo**

Role/Position: Director, Health Emergency Preparedness and Response (HEPR)

Institution: Nigeria Centre for Disease Control and Prevention (NCDC)

Interview Date: 2023

***Part A: Global Health Governance in Nigeria***

**Interviewer:** *How would you rank Nigeria’s leadership in global health? On a scale of 1–10.*

**Dr. Oladejo:** The government has achieved important advancements in bolstering health systems, enhancing surveillance, and improving coordination during health emergencies in cooperation with the NCDC and other partners. However, there is still potential for improvement in Nigeria’s global health governance. The administration has demonstrated a dedication to enhancing health systems, forming alliances, and putting into effect global health standards. Nigeria has taken a leading role in global health governance through participation in international forums, partnerships with international organisations, and adherence to international standards. Issues including resource limitations and capability gaps must still be addressed. I will give a 6 out of 10.

**Interviewer:** *Who are the leading organisations in global health governance in Nigeria?*

**Dr. Oladejo:** In Nigeria, a number of organisations play important roles in governing global health. The Nigeria Centre for Disease Control and Prevention (NCDC) is the principal national organisation in charge of disease surveillance, preparedness, and response. Overall leadership and policy direction are provided by the Federal Ministry of Health. The Nigerian government actively works with foreign organisations like the World Health Organisation (WHO), the United Nations Children’s Fund (UNICEF), and various development partners to improve the health system and respond to public health emergencies.

**Interviewer:** *What actions have the UN, WHO, and other international and national stakeholders taken to improve Nigeria’s healthcare system?*

**Dr. Oladejo:** Major contributions to the improvement of Nigeria’s healthcare system have come from the UN, WHO, and other international and national stakeholders. They have offered technical assistance, funding, resources, and capacity building programmes for the development of human resource capacity, disease surveillance, laboratory strengthening, and healthcare infrastructure. These organisations have also worked with the Nigerian government to create and put into practice health policies, rules, procedures, and plans for medical emergencies, including the COVID-19 pandemic. Their actions have enhanced Nigeria’s capacity for illness detection, rapid response, and general healthcare provision.

***Part B: COVID-19 Pandemic Management in Nigeria (2020–2022)***

**Interviewer:** *What were the precise consequences of the COVID-19 pandemic in Nigeria?*

**Dr. Oladejo:** The COVID-19 pandemic had a profound impact on Nigeria in terms of both socioeconomic and health outcomes. The epidemic spread throughout the nation, resulting in thousands of confirmed cases and fatalities. The pandemic put additional strain on the healthcare system by driving up demand for critical care, treatment, and diagnostic services. The large number of cases created problems with hospital capacity and the availability of healthcare workers. From a socioeconomic perspective, the pandemic caused business interruptions, job losses, and economic downturns. Industries like tourism, hospitality, and small businesses were particularly affected. Social dynamics and daily routines were also impacted by restrictions on movement and public gatherings. Due to school closings and learning disruptions, the pandemic had a significant impact on education. Additionally, there were increased instances of domestic violence and mental health challenges during lockdowns.

**Interviewer:** *What was the nature of the national response to the COVID-19 epidemic, and what partnerships were established?*

**Dr. Oladejo:** The Nigerian government’s comprehensive response to the COVID-19 pandemic was multifaceted and entailed cooperation between a number of parties. To stop the virus from spreading, the government put in place measures such as travel restrictions, lockdowns, and social isolation rules. The coordination of the response, which involved working with the federal and state governments, healthcare organisations, international partners, and community leaders, was largely handled by the NCDC. Public-private partnerships were established to mobilise resources, including funding, testing equipment, and personal protective equipment. The Presidential Task Force on COVID-19 was established by the government through the NCDC to offer high-level coordination and supervision.

**Interviewer:** *What can you name as the key obstacles that Nigeria’s response to the COVID-19 pandemic faced?*

**Dr. Oladejo:** Nigeria experienced a number of difficulties in dealing with the COVID-19 epidemic. These included inadequate healthcare infrastructure and resources, a lack of testing capability, reluctance to receive vaccinations, misinformation, and disregard for preventive actions. Scaling up the response and ensuring equitable access to healthcare treatments were also made difficult by a lack of funding and resources. Our response efforts were also continually hampered by the introduction of new variants and the requirement for continued public awareness and behaviour change.

***Part C: Global Governance During COVID-19***

**Interviewer:** *What precise guidelines and plans were developed globally to manage COVID-19?*

**Dr. Oladejo:** To regulate COVID-19, a variety of policies and methods were developed on a global scale. These included recommendations for testing procedures, contact tracing, quarantine restrictions, infection control measures, vaccination distribution, and public health outreach. These policies and initiatives, founded on best practices and scientific research, were developed and disseminated by international organisations like the WHO.

**Interviewer:** *Which organisation or organisations took the initiative in developing regional intervention policies?*

**Dr. Oladejo:** During the COVID-19 pandemic, the World Health Organisation (WHO) was instrumental in developing regional plans for intervention. They devised protocols, gave technical advice, and helped member governments share information. In the context of Africa, the African Union’s Africa Centres for Disease Control and Prevention (Africa CDC) also made a significant contribution to the creation and coordination of regional policy.

**Interviewer:** *How were these plans and tactics put into action?*

**Dr. Oladejo:** Strong cooperation between international, regional, and national players was necessary for the execution of these policies and programmes. Member governments received technical assistance, training, and capacity building support from the WHO and other international partners. Through their respective health organisations and emergency response systems, national governments, including Nigeria, implemented these policies after adapting them to their specific local settings. The effectiveness of the strategies was evaluated regularly, and necessary revisions were made.

**Interviewer:** *What part did Nigeria play in this process?*

**Dr. Oladejo:** During the COVID-19 pandemic, Nigeria actively participated in the global governance process. The nation participated in discussions about policy formulation and coordinated its response efforts with international standards. The NCDC and other Nigerian health officials actively participated in international forums where they shared experiences, best practices, and lessons learned. Nigeria worked in tandem with other regional nations and contributed to the implementation of regional plans for the West African subregion.

***Part D: National Initiatives in the COVID-19 Era***

**Interviewer:** *Have there been any appreciable advancements in national thinking and governance since the COVID-19 pandemic erupted in the areas of policy guidance, intelligence and oversight, system design, collaboration and coalition building, and accountability?*

**Dr. Oladejo:** Yes, the COVID-19 pandemic has significantly improved the way the country thinks and governs. The epidemic brought attention to the need for more effective and flexible public health regulations. Nigeria has improved its framework for responding to public health emergencies, created national COVID-19 guidelines, and implemented procedures for disease surveillance, contact tracing, and data management. There has been a greater emphasis on monitoring systems and early illness outbreak identification in terms of intelligence and oversight. Through the NCDC, the Nigerian government has enhanced its capacity for surveillance, data gathering, and analysis, improving the nation’s capacity to track and address public health threats promptly and efficiently. The COVID-19 pandemic revealed flaws in Nigeria’s healthcare system and supply chain management. Efforts are being made to improve medical supply availability and delivery, increase testing capacity, strengthen laboratory services, and reinforce healthcare systems. Collaboration and coalition building have also improved, with better coordination between government entities, non-governmental organisations, development partners, and local leaders. This has facilitated the sharing of materials, knowledge, and best practices in the fight against COVID-19. Lastly, accountability systems have been improved to guarantee openness and effective resource management. The NCDC, the Presidential Task Force on COVID-19, and other relevant organisations are responsible for managing funding, tracking expenditures, and reporting on the utilisation of resources in the pandemic response.

**Interviewer:** *What contributions, if any, do international players provide to these new national initiatives?*

**Dr. Oladejo:** International organisations and other global actors have offered assistance in the form of finance, technical support, capacity building, and resource mobilisation. Nigeria’s national programmes have received significant support from the WHO, the Africa CDC, and development partners like the World Bank and the United Nations. They have offered direction, promoted knowledge transfer, and directed financial resources to improve Nigeria’s response capability.

**Interviewer:** *How do you believe Nigeria can create a long-lasting national framework for health governance?*

**Dr. Oladejo:** It takes a comprehensive strategy to create a sustainable national framework for health governance. Infrastructure investments in healthcare, human resources, and research and development must be given top priority in Nigeria. The primary healthcare system must be strengthened, illness surveillance and response skills enhanced, and universal health coverage made a priority. Additionally, it is essential for the government, commercial sector, and civil society organisations to work together. Public-private partnerships can support innovative healthcare delivery, technology transfer, and sustainable finance systems. A sustainable national framework for health governance will also be developed through ongoing capacity building, information exchange, and learning from international best practices.

**Interview 7: Dr. Oyeladun Okunromade**

Role/Position: Director, Surveillance and Epidemiology; Head, International Health Regulations National Focal Point (NFP); COVID-19 National Surveillance Coordinator

Institution: Nigeria Centre for Disease Control and Prevention (NCDC)

Interview Date: 2023

***Part A: Global Health Governance in Nigeria***

**Interviewer:** *How would you rate the current state of global health governance in Nigeria? On a scale of 1–10.*

**Dr. Okunromade:** Global health governance in Nigeria has seen significant improvements in recent years. We have strengthened our collaborations with various organisations and stakeholders to enhance our response to public health emergencies. On a scale, I will say 7 out of 10.

**Interviewer:** *Which organisations are prominent in global health governance in Nigeria?*

**Dr. Okunromade:** The World Health Organisation (WHO) plays a crucial role in global health governance in Nigeria. Additionally, we have partnerships with other international organisations such as the United Nations, the Africa Centres for Disease Control and Prevention (Africa CDC), and various donor agencies.

**Interviewer:** *In what ways have the United Nations, WHO, and other global and national stakeholders intervened in strengthening the health system in Nigeria?*

**Dr. Okunromade:** The United Nations, WHO, and other stakeholders have provided technical assistance, capacity building, and financial support to strengthen our health system. They have helped in areas such as surveillance, laboratory diagnostics, infection prevention and control, and training of healthcare workers, as well as supplying critical needs during the pandemic.

***Part B: COVID-19 Pandemic Management in Nigeria (2020–2022)***

**Interviewer:** *What were the specific effects of the COVID-19 pandemic in Nigeria?*

**Dr. Okunromade:** The COVID-19 pandemic had significant impacts on Nigeria. We experienced an increase in confirmed cases, hospitalisations, and deaths. The pandemic also had socio-economic consequences, affecting various sectors such as education, trade, and transportation. Businesses suffered due to reduced patronage, and other associated challenges.

**Interviewer:** *What was the nature of the national response, and what collaborations were forged in response to the COVID-19 pandemic?*

**Dr. Okunromade:** The national response to the COVID-19 pandemic was multifaceted. We established emergency operation centres, implemented testing and contact tracing, enforced public health measures, and conducted public awareness campaigns. We collaborated with various stakeholders, including the federal and state governments, private sector organisations, and international partners.

**Interviewer:** *What can you identify as the main challenges militating against the response to the COVID-19 pandemic in Nigeria?*

**Dr. Okunromade:** There were several challenges we faced during the response. Limited testing capacity, inadequate healthcare infrastructure, misinformation and conspiracy theories, and compliance issues with public health guidelines were among the key challenges. Additionally, vaccine distribution challenges and hesitancy posed significant hurdles.

***Part C: Global Governance During COVID-19***

**Interviewer:** *What specific policies and strategies were formulated at the global level to control COVID-19?*

**Dr. Okunromade:** At the global level, policies and strategies were developed to guide countries in controlling COVID-19. These included recommendations for testing, contact tracing, quarantine measures, travel restrictions, and vaccine distribution.

**Interviewer:** *Which organisation or organisations took the lead in the formulation of regional policies for intervention?*

**Dr. Okunromade:** The World Health Organisation (WHO) played a leading role in formulating regional policies and providing technical guidance to member states. They worked closely with other international organisations and expert groups to develop evidence-based recommendations.

**Interviewer:** *How were these policies and strategies implemented?*

**Dr. Okunromade:** The implementation of global policies and strategies involved collaboration between the WHO and national health authorities. The WHO provided technical support, training, and resources to countries, including Nigeria, to implement the recommended measures.

**Interviewer:** *What was Nigeria’s role in this process?*

**Dr. Okunromade:** Nigeria actively participated in global discussions and contributed to the development of policies and strategies. We aligned our national response with the global guidelines and collaborated with the WHO and other stakeholders to implement them effectively.

**Interviewer:** *What was the level of intelligence gathering and surveillance at the national level to control COVID-19?*

**Dr. Okunromade:** Nigeria strengthened its surveillance systems to detect and monitor COVID-19 cases. We enhanced laboratory testing capacity, implemented active case finding, and established a robust surveillance network to track the spread of the virus.

**Interviewer:** *Which organisation or state was responsible for oversight in terms of the global and national response?*

**Dr. Okunromade:** The Nigeria Centre for Disease Control and Prevention (NCDC) was responsible for coordinating and overseeing the national response to COVID-19. We worked in close collaboration with the Federal Ministry of Health and other relevant agencies.

**Interviewer:** *How was collaboration among Nigeria and neighbouring countries in the COVID-19 response?*

**Dr. Okunromade:** Collaboration among Nigeria and neighbouring countries was crucial in the COVID-19 response. We established regional partnerships and information-sharing mechanisms to coordinate surveillance efforts, border controls, and response strategies. This collaboration helped in identifying and managing cross-border transmission risks.

**Interviewer:** *How were local and international NGOs and civil society groups involved in the COVID-19 response?*

**Dr. Okunromade:** Local and international NGOs, as well as civil society groups, played a vital role in the COVID-19 response. They provided support in areas such as community engagement, risk communication, distribution of relief materials, and advocacy for public health measures.

**Interviewer:** *Were there any non-coalition or public-private partnerships formed to control and contain COVID-19 at the national level?*

**Dr. Okunromade:** Yes, several non-coalition and public-private partnerships were formed to control and contain COVID-19 in Nigeria. These partnerships involved collaborations between the government, private sector organisations, philanthropic foundations, and civil society groups to mobilise resources, support healthcare infrastructure, and enhance the response. One very important coalition was the CA-COVID group, which committed substantial resources to ending the scourge of the pandemic.

**Interviewer:** *How were human and material resources pooled at the national level in the response to COVID-19?*

**Dr. Okunromade:** Pooled resources were essential in the national response to COVID-19. The government allocated funds to the health sector, and international partners provided financial and material support. We also mobilised human resources, including healthcare workers, volunteers, and experts, to strengthen the response.

**Interviewer:** *What specific national initiatives helped in the country’s containment of COVID-19?*

**Dr. Okunromade:** Nigeria implemented various national initiatives to contain COVID-19. These included establishing isolation and treatment centres, implementing public health measures such as mask mandates and social distancing, conducting mass testing campaigns, and accelerating the vaccination rollout. The CA-COVID group was also particularly helpful in this regard.

**Interviewer:** *Was any national organisation charged with the responsibility of managing resources in response to COVID-19?*

**Dr. Okunromade:** Yes, the Presidential Task Force on COVID-19, chaired by the Secretary to the Government of the Federation, was responsible for managing resources and coordinating the national response.

**Interviewer:** *How were those funds managed, in your opinion?*

**Dr. Okunromade:** The funds allocated for the COVID-19 response were managed through transparent processes, in accordance with existing financial regulations and guidelines. The Presidential Task Force ensured accountability and oversight in the management of these resources.

**Interviewer:** *In terms of Nigeria’s contribution to the overall global response to COVID-19, what can you tell us?*

**Dr. Okunromade:** Nigeria made significant contributions to the global response to COVID-19. We shared our experiences, best practices, and lessons learned from similar outbreaks with other countries. Additionally, Nigerian scientists and researchers actively participated in global research efforts and contributed to the development of diagnostic tools and treatment protocols.

**Interviewer:** *What is your assessment of Nigeria’s role in the response to COVID-19?*

**Dr. Okunromade:** Nigeria played a critical role in the response to COVID-19. Despite the challenges we faced, we demonstrated resilience, adaptability, and strong leadership. Our national response efforts were commended by international partners, and we continue to work towards strengthening our healthcare system to better prepare for future pandemics.

***Part D: National Initiatives in the COVID-19 Era***

**Interviewer:** *Have there been any noticeable improvements in national thinking and governance since the COVID-19 pandemic erupted in the areas of policy guidance, intelligence and oversight, system design, collaboration and coalition building, and accountability?*

**Dr. Okunromade:** Yes, the COVID-19 pandemic has served as a catalyst for improvements in national thinking and governance. We have seen enhanced policy guidance and coordination, strengthened surveillance systems, improved healthcare infrastructure, and increased collaboration among stakeholders. Accountability mechanisms have also been strengthened to ensure transparency in the management of resources.

**Interviewer:** *What are the global actors’ contributions to these new national initiatives, if any?*

**Dr. Okunromade:** Global actors, including international organisations, donor agencies, and technical partners, have provided support in terms of technical expertise, financial resources, and capacity building. Their contributions have been instrumental in driving these new national initiatives and strengthening our health governance framework.

**Interviewer:** *How do you suggest Nigeria develops a sustainable national framework for health governance?*

**Dr. Okunromade:** Developing a sustainable national framework for health governance requires a multi-sectoral approach. We need to strengthen partnerships between the government, private sector, civil society, and communities. Investing in healthcare infrastructure, human resources, research and development, and continuous capacity building will be crucial in building a resilient health system that can effectively respond to future health crises.

**NOTE ON INTERVIEW GUIDE AND TRANSCRIPT COMPLETENESS**

Seven in-depth interviews were conducted and audio-recorded for this study. All seven interviews have been transcribed and are presented in full in this supplementary file. The transcripts have been lightly edited for clarity: filler words, false starts, technical interruptions, and off-topic exchanges have been removed. The substantive content of all responses has been preserved in full.

Interviews 1–4 (Prof. Rosemary Audu, Dr. Segun Adeoye, Dr. Bowale Abimbola, and Dr. Dapo Asiyanbi) were sourced from the original audio recordings. Interviews 5–7 (Dr. Chinwe Ochu, Dr. John Oladejo, and Dr. Oyeladun Okunromade) have been updated with full written transcripts subsequently provided by the respective interviewees following the original recording sessions.

The interview guide used across all sessions was consistent and is original to this study, developed as part of the corresponding author’s PhD thesis at Covenant University. The guide was structured around four thematic parts guided by the World Health Organisation’s Framework for Strengthening Health Systems to Improve Health Outcomes (2007). Data saturation was confirmed at the point of seven interviews.
