# Supplementary material for: National health institutions and the management of COVID-19 pandemic: fulfilling the sustainable development goal three (SDG-3) in Nigeria
Source: BMC Public Health. 2026 Aug 7;26:3189. doi: 10.1186/s12889-026-28896-8 (PMC13733176; doi:10.1186/s12889-026-28896-8)
Supplement: Supplementary file 2 [file 12889_2026_28896_MOESM2_ESM.docx]

**COREQ CHECKLIST - National Health Institutions and the Management of COVID-19 Pandemic: Fulfilling the Sustainable Development Goal Three (SDG-3) in Nigeria**

| **Item No.** | **Guide Questions/Description** | **Reported Response (from your Manuscript)** | **Page No.** |
| --- | --- | --- | --- |
| **Domain 1: Research team and reflexivity** |  |  |  |
| **1** | **Which author/s conducted the interview?** | **Gideon I. Folorunso conducted the field interviews.** | **10** |
| **2** | **What were the researcher’s credentials?** | **BSc, MSc, PhD (Department of Political Science and International Relations).** | **N/A** |
| **3** | **What was their occupation at the time of the study?** | **Academic Researcher / Scholar at Covenant University.** | **N/A** |
| **4** | **Was the researcher male or female?** | **Male** | **N/A** |
| **5** | **What experience or training did the researcher have?** | **GIF was supervised by MMD and FCC.** | **10** |
| **6** | **Was a relationship established prior to study commencement?** | **No** | **N/A** |
| **7** | **What did the participants know about the researcher?** | **Participants were informed the research was for academic purposes and gave informed consent.** | **4** |
| **8** | **What characteristics were reported about the interviewer/facilitator?** | **The authors declare that they have no competing interests.** | **10** |
| **Domain 2: Study design** |  |  |  |
| **9** | **What methodological orientation was stated to underpin the study?** | **Retrospective qualitative research / WHO Framework for Strengthening Health Systems.** | **4** |
| **10** | **How were participants selected?** | **Purposive sampling.** | **4** |
| **11** | **How were participants approached?** | **Virtually via teleconferencing, and physically.** | **4** |
| **12** | **How many participants were in the study?** | **Seven (7) participants.** | **4** |
| **13** | **How many people refused to participate or dropped out?** | **Not reported.** | **N/A** |
| **14** | **Where was the data collected?** | **Virtually via teleconferencing, and live recording.** | **4** |
| **15** | **Was anyone else present besides the participants and researchers?** | **No** | **N/A** |
| **16** | **What are the important characteristics of the sample?** | **Top-level directors from NCDC, NIMR, WAHO, Lagos State COVID-19 Case Mgt. Team, and LSPHCB.** | **4** |
| **17** | **Were questions, prompts, guides provided by the authors? Was it pilot tested?** | **An interview guide based on the WHO Framework for Action (2007) was used.** | **4** |
| **18** | **Were repeat interviews carried out?** | **No** | **N/A** |
| **19** | **Did the research use audio or visual recording to collect the data?** | **Audio recording** | **4** |
| **20** | **Were field notes made?** | **Yes.** | **N/A** |
| **21** | **What was the duration of the interviews?** | **30-40 minutes** | **N/A** |
| **22** | **Was data saturation discussed?** | **Yes, data saturation was the basis for determining the sample size.** | **4** |
| **23** | **Were transcripts returned to participants for comment and/or correction?** | **No** | **N/A** |
| **Domain 3: analysis and findings** |  |  |  |
| **24** | **How many data coders coded the data?** | **One primary coder (GIF); MMD and FCC supervised methodology and data interpretation.** | **10** |
| **25** | **Did authors provide a description of the coding tree?** | **A thematic diagram mapping the framework is provided (Figure 2).** | **5** |
| **26** | **Were themes identified in advance or derived from the data?** | **Themes were guided by the WHO Framework.** | **5** |
| **27** | **What software, if applicable, was used to manage the data?** | **Gephi.** | **N/A** |
| **28** | **Did participants provide feedback on the findings?** | **Yes.** | **5-9** |
| **29** | **Were participant quotations presented? Was each quotation identified?** | **Yes, identified by institutional role (e.g., "The NIMR respondent emphasised...").** | **5-9** |
| **30** | **Was there consistency between the data presented and the findings?** | **Yes.** | **5-9** |
| **31** | **Were major themes clearly presented in the findings?** | **Yes (Challenges, Responses and Collaborations, Measures for Future Preparedness).** | **5-9** |
| **32** | **Is there a description of diverse cases or discussion of minor themes?** | **Yes, minor themes like socio-economic unrest (#ENDSARS) and the "infodemic" are discussed.** | **5-6** |

Developed from: Tong A, Sainsbury P, Craig J. Consolidated criteria for reporting qualitative research (COREQ): a 32-item checklist for interviews and focus groups. *International Journal for Quality in Health Care*. 2007. Volume 19, Number 6: pp. 349 – 357
